# Advances in myopia research anatomical findings in highly myopic eyes

**DOI:** 10.1186/s40662-020-00210-6

**Published:** 2020-09-02

**Authors:** Jost B. Jonas, Ya Xing Wang, Li Dong, Yin Guo, Songhomitra Panda-Jonas

**Affiliations:** 1 Department of Ophthalmology, Medical Faculty Mannheim of the Ruprecht-Karis-University, Universitäts-Augenklinik, Theodor-Kutzer-Ufer 1-3, 68167 Mannheim, Germany; 2 Beijing Ophthalmology and Visual Sciences Key Laboratory, Beijing Institute of Ophthalmology, Beijing Tongren Hospital, Capital Medical University, Beijing, China; 3 Beijing Tongren Eye Center, Beijing Ophthalmology and Visual Science Key Lab, Beijing Key Laboratory of Intraocular Tumor Diagnosis and Treatment, Beijing Tongren Hospital, Capital Medical University, Beijing, China; 4 Tongren Eye Care Center, Beijing Tongren Hospital, Capital Medical University, Beijing, China

**Keywords:** High myopia, Myopia, Bruch’s membrane, Optic nerve head, Optic disc, Parapapillary gamma zone, Parapapillary delta zone, Lamina cribrosa, Anatomical changes

## Abstract

**Background:**

The goal of this review is to summarize structural and anatomical changes associated with high myopia.

**Main text:**

Axial elongation in myopic eyes is associated with retinal thinning and a reduced density of retinal pigment epithelium (RPE) cells in the equatorial region. Thickness of the retina and choriocapillaris and RPE cell density in the macula are independent of axial length. Choroidal and scleral thickness decrease with longer axial length in the posterior hemisphere of the eye, most marked at the posterior pole. In any eye region, thickness of Bruch’s membrane (BM) is independent of axial length. BM opening, as the inner layer of the optic nerve head layers, is shifted in temporal direction in moderately elongated eyes (axial length <26.5 mm). It leads to an overhanging of BM into the intrapapillary compartment at the nasal optic disc side, and to an absence of BM at the temporal disc border. The lack of BM at the temporal disc side is the histological equivalent of parapapillary gamma zone. Gamma zone is defined as the parapapillary region without BM. In highly myopic eyes (axial length >26.5 mm), BM opening enlarges with longer axial length. It leads to a circular gamma zone. In a parallel manner, the peripapillary scleral flange and the lamina cribrosa get longer and thinner with longer axial length in highly myopic eyes. The elongated peripapillary scleral flange forms the equivalent of parapapillary delta zone, and the elongated lamina cribrosa is the equivalent of the myopic secondary macrodisc. The prevalence of BM defects in the macular region increases with longer axial length in highly myopic eyes. Scleral staphylomas are characterized by marked scleral thinning and spatially correlated BM defects, while thickness and density of the choriocapillaris, RPE and BM do not differ markedly between staphylomatous versus non-staphylomatous eyes in the respective regions.

**Conclusions:**

High axial myopia is associated with a thinning of the sclera and choroid posteriorly and thinning of the retina and RPE density in the equatorial region, while BM thickness is independent of axial length. The histological changes may point towards BM having a role in the process of axial elongation.

## Background

Axial myopia is characterized by an elongation of the sagittal diameter of the eye [1]. After birth, the eyes grow spherically and enlarge from a diameter of approximately 17 mm to a diameter of about 21 to 22 mm roughly at the end of the second year of life. At that time, the cornea and lens have assumed almost adult proportions. In the following years, the optical axis elongates and adapts to the optical properties of the cornea and lens so that eventually, in the ideal case, an emmetropic stage develops [2]. If the optical axis becomes longer than needed for emmetropia, axial myopia results. Since the pathophysiological mechanisms underlying the process of emmetropization and myopization have not been completely elucidated yet, the assessment of anatomical features and changes associated with axial myopia may be of interest to further uncover the mechanisms involved [1, 3]. The study presents recent histological findings and is additionally and partially based on a literature search. This search targeted English-language articles in PubMed spanning all dates, with the general search terms of optic disc, optic nerve head, myopia, high myopia, histology, gamma zone, delta zone, parapapillary atrophy, Bruch’s membrane and lamina cribrosa. This study is an extension of a previous review on a similar topic [4].

## Main Text

### Sclera

Studies examining enucleated human globes have suggested that up to the second year of life, the volume of the sclera increases, while beyond that age, the scleral volume is not changed substantially [5, 6]. This indicates that the axial elongation and associated scleral thinning observed beyond two years of age may be related to the rearranging of available scleral tissue rather than formation of additional scleral tissue [7–9]. It may point against a primary active role of the sclera in the process of emmetropization and axial elongation. The thinning of the sclera in association with the axial globe enlargement was most marked at the posterior pole and least marked in the ora serrata [7–9]. It indicated that scleral changes in association with primary myopia occur mostly posterior to the ora serrata. These observations made in human globes concurred with findings made in a study by McBrien et al. who reported on a significant scleral thinning and scleral tissue loss, particularly at the posterior pole, in young tree shrews with induced myopia [10]. After a period of 12 days of myopia induction, the collagen fibril diameter distribution was not significantly altered, while after a period of 3-20 months of myopia induction, significant reductions in the collagen fibril diameter were found, particularly at the posterior pole. McBrien and colleagues concluded that a loss of scleral tissue and subsequent scleral thinning occurred rapidly during development of axial myopia, while an increased number of small diameter collagen fibrils in the sclera of highly myopic eyes was observed only in the longer term.

### Choroid

In a similar manner, histomorphometric investigations suggested that also the choroidal volume in adolescents and adults, including adults with extreme axial myopia, was not related with axial length [6]. Although one has to take into account the limitations of histomorphometric studies with marked post-mortem changes in the choroidal space, the findings suggest that the axial elongation and associated choroidal thinning, observed in adolescents and adults by histomorphometry and *in-vivo* by optical coherence tomography, may be related to a rearranging of available choroidal tissue rather than formation of additional choroidal tissue [6].

### Bruch’s membrane

In contrast to the scleral and choroidal thickness, the thickness of Bruch’s membrane (BM) was independent of axial length which may indicate that its volume increased with higher myopia [11–13]. BM thickness at the posterior pole was similar in eyes with an axial length of >30 mm and in eyes with an axial length of 24 mm. It indicates that extremely highly myopic eyes and emmetropic eyes have the same thickness of BM, in particular at the posterior pole. Interestingly, eyes with secondary high myopia due to congenital glaucoma showed a thinning of BM with longer axial length [14, 15].

Parallel to the finding that BM thickness was independent of axial length, also the retinal thickness in the foveola, as measured by optical coherence tomography, and the density of the retinal pigment epithelium (RPE) cells in the macular region, as measured by histomorphometry, were not related to axial length [16, 17]. The thickness of the fovea in highly myopic eyes (without myopic maculopathy) was similar to the foveal thickness in emmetropic eyes. Interestingly, in the fundus periphery in the retro-equatorial and equatorial region, the density of the RPE and the thickness of the retina decreased with longer axial length [16, 17]. Again, in contrast to globes with primary myopia, eyes with secondary high myopia due congenital glaucoma showed a decreasing density of the macular RPE cells with longer axial length [14, 18].

### Bruch’s membrane and process of elongation

Taking into account that the process of emmetropization refers to the length of the optical axis (which ends at the photoreceptor outer segments close to BM), it would make sense to consider BM as the primary driver for the axial elongation of the eye [19]. Conversely, the sclera is separated from the photoreceptor outer segments by the spongy choroid with a thickness of approximately 250 μm, with the choroidal thickness in addition showing a dependence on the daytime [20]. If one considers that the process of emmetropization occurs with a precision of about 100 μm of axial length (with 300 μm in axial length representing one diopter of ametropia), BM as compared to the sclera appears to be better suited as the structure elongating the eye. Correspondingly, a recent investigation demonstrated that BM had a relatively high biomechanical strength. The average elastic (tangent) moduli of BM samples at 0% and 5% strain were 1.60 ± 0.81 MPa and 2.44 ± 1.02 MPa, respectively. Burst tests demonstrated that BM could withstand an intraocular pressure (IOP) of on average 82 mmHg before rupturing [21]. Considering BM instead of the sclera as the primary mover for the elongation of the eye could also explain the choroidal thinning in myopic eyes [22]. If the sclera were the structure causing the eye to be longer, the choroidal space would have become wider [19].

It has to be pointed out that the hypothesis of BM as the primary mover for ocular elongation has not been confirmed and that various previous investigations have pointed to the choroid and/or sclera as the tissue layers with an active role in regulating the axial growth of the eye and primarily making the eye longer. To cite examples, Marzani and Wallman have provided compelling evidence showing how the choroid influences the proteoglycan synthesis of the sclera in chick eyes undergoing and recovering from form-deprivation [23]. There is also evidence from humans suggesting an active contribution of the choroid in the process of emmetropization [24, 25]. Not focusing on the choroid and sclera in this review does not mean at all, that they may not be involved in the process of myopization. It also includes the influence of wavelength on emmetropization as shown in many experimental studies, and the influence of defocus of images on the process of axial elongation [26–39]. The findings of some of these studies would clearly not favor a pressure from Bruch’s membrane leading to a compression of the posterior choroid and an expansion of the posterior sclera. With respect to diurnal changes in choroidal thickness in the discussion of the process of axial elongation, one may also consider that there is evidence for these diurnal rhythms of choroidal thickness potentially contributing to diurnal changes in scleral proteoglycan synthesis, and regulation of axial eye growth, at least in chicks [40, 41]. It shows that the elucidation of the mechanism of axial elongation in myopic eyes is far from being complete and that the hypothesis of BM as a major driver in the process is far from being proven.

**Fig. 1:**
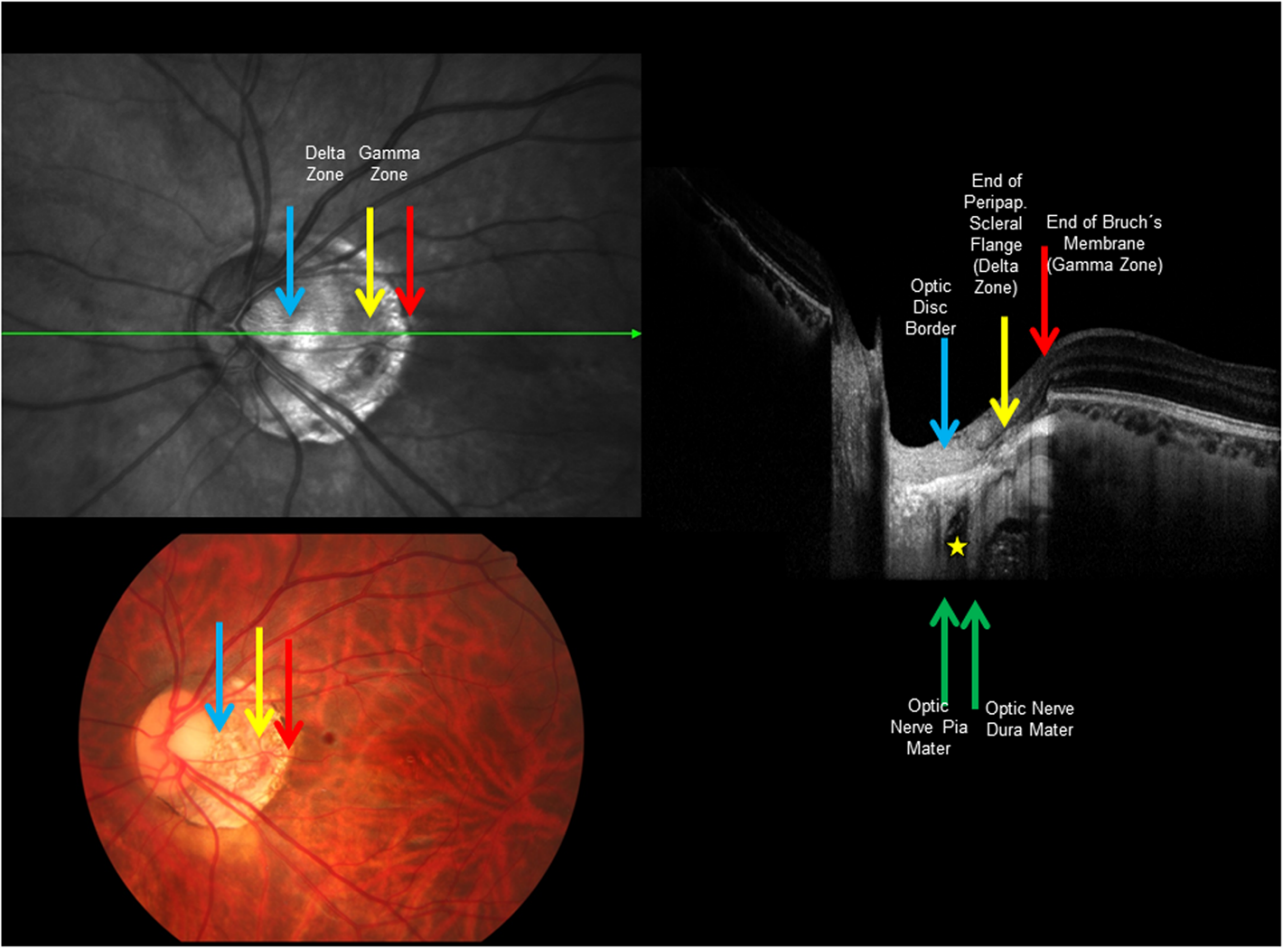
Fundus photograph and optical coherence tomographic images of the same highly myopic eye with parapapillary gamma and delta zones. Blue arrow: optic disc border; yellow arrows: border between delta zone and gamma zone; red arrows: outer border of gamma zone; green arrows: optic nerve meninges (pia mater and dura mater); yellow asterisk: orbital cerebrospinal fluid space

**Fig. 2:**
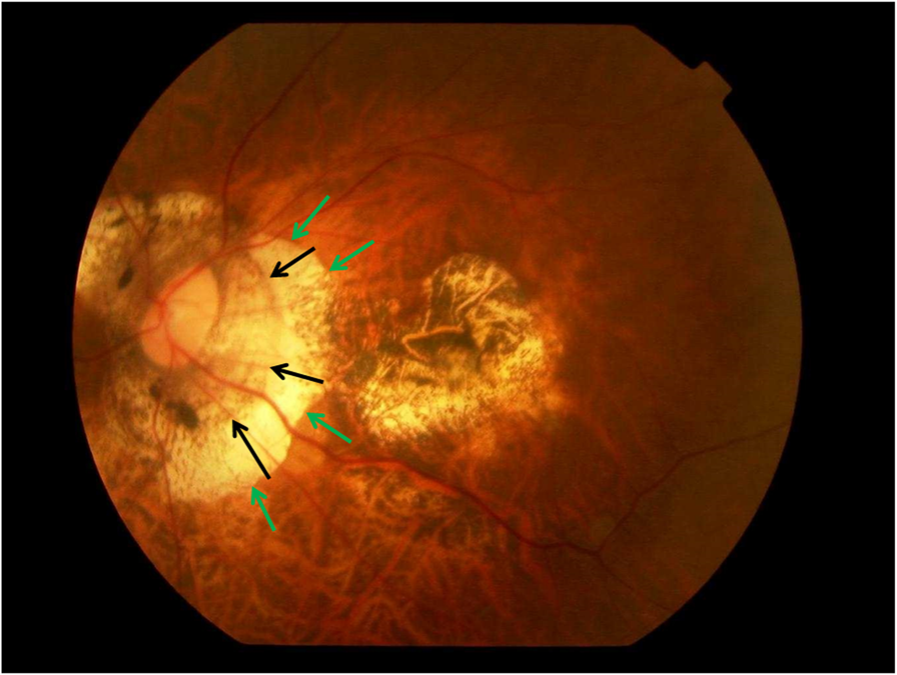
Fundus photograph of a highly myopic eye with myopic maculopathy category 4 (macular atrophy or macular Bruch’s membrane defect), and with parapapillary gamma zone (green arrows) and delta zone (black arrows)

If BM is actively involved in the process of axial elongation, one may assume that it is produced in the equatorial and retro-equatorial regions, leading to an increase in the sagittal diameter of the globe by pushing the BM at posterior pole backwards [19]. It would lead to a compression and thinning of the choroid and secondary to a thinning of the sclera, most marked at the posterior pole. The process of axial elongation affects the sagittal globe diameter the most, while the horizontal and vertical globe diameters enlarge only slightly by about 0.1 to 0.2 mm for each mm increase in axial length [42]. This increase in the coronal diameters of the eye leads to an increase in the optic disc fovea distance [43, 44]. Studies have suggested that the axial elongation-associated increase in the disc-fovea distance is due to the development and enlargement of gamma zone in the temporal parapapillary region (Figs. 1 and 2) [43, 45, 46]. It corroborates with histological studies and population-based investigations that showed that the length of the macular BM as measured by optical coherence tomography (OCT) was independent of axial length, while the length of the peripapillary border tissue of the choroid as measured histomorphometrically increased with longer axial length [47, 48]. The peripapillary border tissue of the choroid connects the end of BM with the peripapillary border tissue of the peripapillary scleral flange and with the lamina cribrosa [48]. The finding, that BM at the posterior pole did not elongate with longer axial length, could explain the observations that BM thickness, the density of macular RPE cells, the foveal retinal thickness and the thickness and density of the choriocapillaris were independent of axial elongation [11–13, 16, 17]. It could also explain that best corrected visual acuity in myopic eyes without myopic maculopathy was independent of axial length [49]. The enlargement of BM in the fundus periphery could explain the decrease in the RPE cell density in that region since RPE cells, without increasing their number, would have to spread over a larger area [17]. Correspondingly, the thinning of the peripheral retina in eyes with elongating axis could be explained by the larger area the retina has to cover in the fundus periphery [16]. The notion of BM expanding in the fundus periphery and causing the axial elongation of the eye fits with the hypothesis that the sensory part of the feedback mechanism in the process of emmetropization has been assumed to be located in the fundus periphery [29, 50–52].

### Optic nerve head

**Fig. 3:**
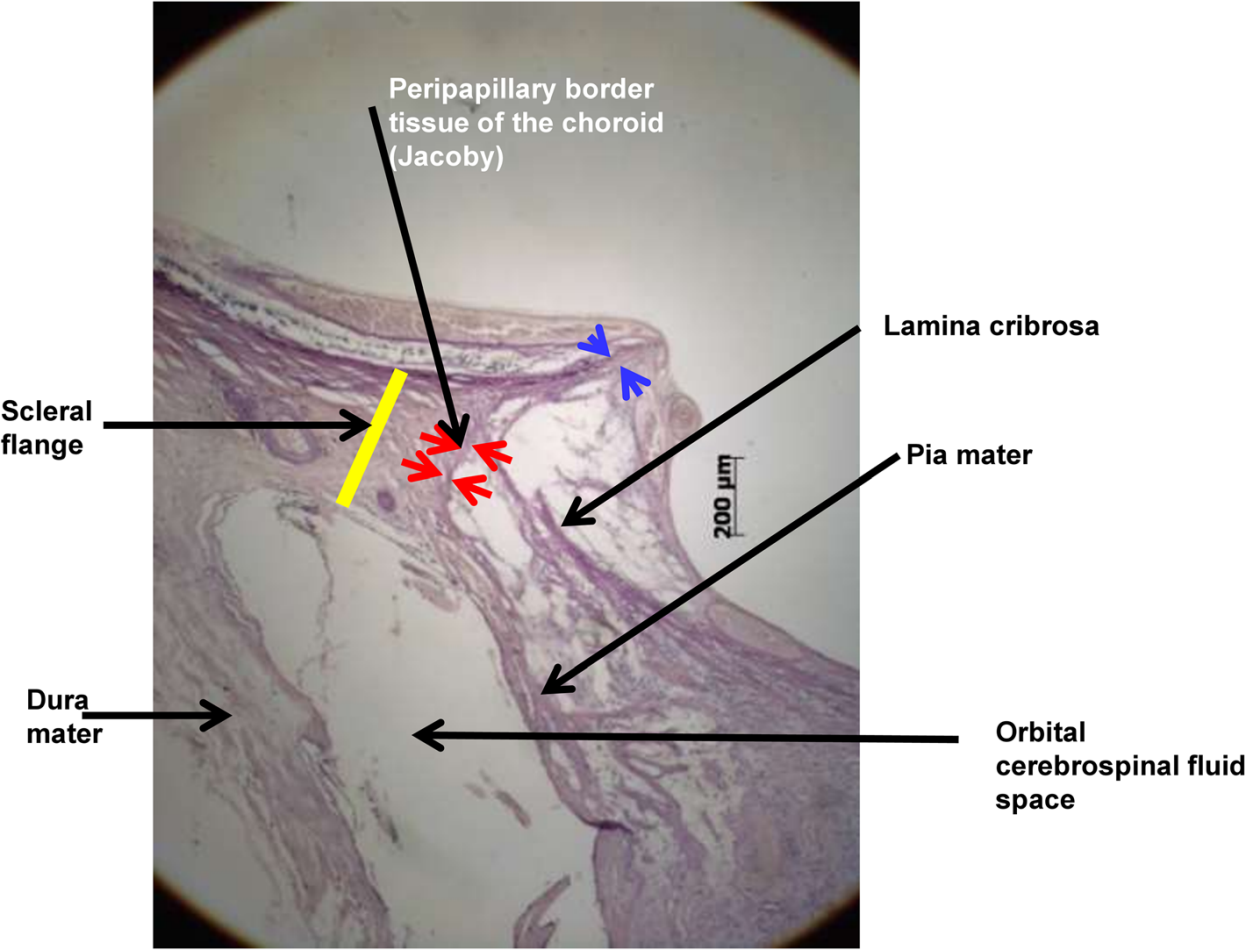
Histophotograph of the optic disc border of a glaucomatous highly myopic eye in which the peripheral part of the extended lamina cribrosa is not buffered by solid optic nerve tissue but faces, covered by optic nerve pia mater, the orbital cerebrospinal fluid space

The axial elongation in high myopia leads to marked changes in the anatomy of the optic nerve head [46, 53]. Longer axial length is correlated with an enlargement of the optic disc, defined as the area with the lamina cribrosa as bottom [54, 55]. The enlarged disc is also called secondary or acquired macrodisc. The enlargement of the optic disc leads to an elongation, presumably stretching, and thinning of the lamina cribrosa [55, 56]. Parallel to the stretching of the lamina cribrosa, the optic cup gets flattened, so that the spatial contrast between the height of the neuroretinal rim and the depth of the optic cup as seen upon ophthalmoscopy is reduced [46, 55]. The lamina cribrosa thinning geometrically leads to a decreased distance between the intraocular compartment with the IOP and the retrobulbar compartment which is the orbital cerebrospinal fluid (CSF) space with the orbital CSF pressure [57]. Both, the IOP and the CSF pressure are the determinants of the trans-lamina cribrosa pressure difference that exerts force on the retinal ganglion cell axons when passing through the lamina cribrosa [56, 58, 59]. If the IOP and CSF pressure are unchanged but if the distance between both compartments is reduced, the trans lamina cribrosa pressure gradient gets steepened which may be one of the reasons for an increased glaucoma susceptibility in highly myopic eyes [60, 61]. By the enlargement of the posterior surface of the lamina cribrosa, only the central region of the lamina cribrosa is buffered by the solid tissue of the optic nerve, while the annular peripheral region of the posterior lamina cribrosa surface has direct contact with the CSF space (Fig. 3). It allows a backward bowing of the peripheral lamina cribrosa into the widened orbital CSF space in highly myopic eyes, which may be an additional reason for the increased glaucoma susceptibility in high myopia. Such a backward bowing of the lamina cribrosa in highly myopic can be better detected by OCT than by ophthalmoscopy. In non-highly myopic patients, a localized backward bowing of the lamina cribrosa leads to so called acquired pits of the optic nerve head, as described by Spaeth et al. (“APON”) [62].

**Fig. 4:**
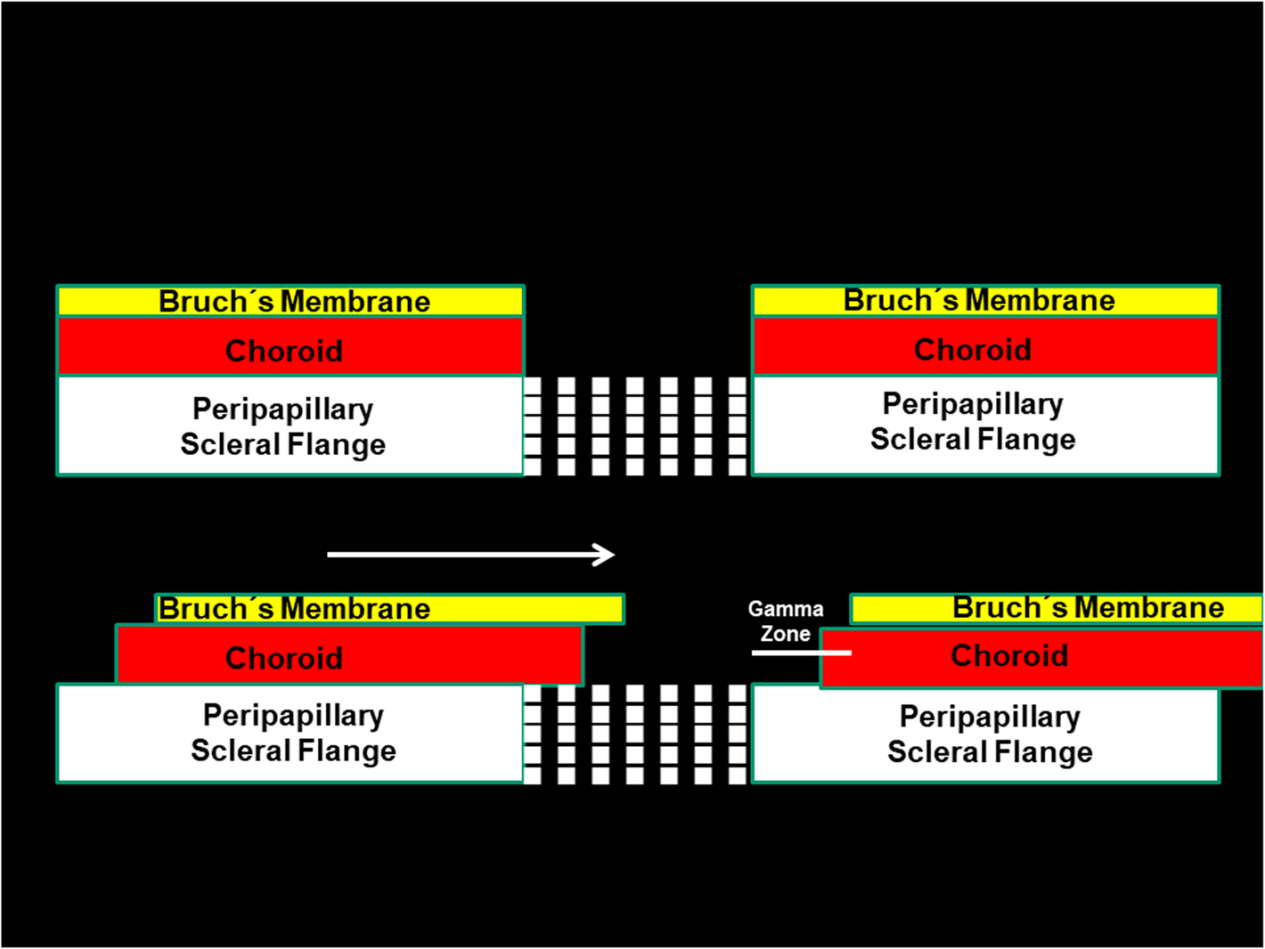
Scheme illustrating the three layers of the optic nerve head and a shift of Bruch’s membrane (BM) opening, leading to an overhanging of BM into the intrapapillary compartment on one side and a corresponding lack of BM on the other side (gamma zone)

Histologically, the optic nerve head can be compared with a three-layered hole, with the BM opening as its inner layer, the choroidal opening as the middle layer, and the peripapillary scleral flange opening, covered by the lamina cribrosa, as its outer layer [63]. At birth, all three layers are mostly aligned to each other. In adolescents with increased myopia, the BM opening may shift in the direction of the posterior pole, potentially caused by the production of BM in the fundus periphery. Since the sclera is not firmly connected to the choroid (except for the scleral spur anteriorly, the exit of the vortex veins, and the peripapillary border tissue of the choroid posteriorly), the scleral opening of the optic disc may move less than the BM opening does. It leads to an oblique exit canal for the retinal ganglion cell axons from the posterior pole in nasal anterior direction before, when leaving the canal, bending backwards and running towards the upper nasal part of the orbital apex. The temporal shift of BM opening leads to an overhanging of the edge of BM opening into the intrapapillary compartment at the nasal disc side, and correspondingly, to a lack of BM at the temporal disc side [63, 64]. It is the basis for the development of parapapillary gamma zone, which is defined by the lack of BM and which is usually located at the temporal to temporal inferior parapapillary region (Figs. 1, 2, 3 and 4) [65–72]. In eyes with a so called tilted optic disc, the BM opening shifted mostly inferiorly, leading to an overhanging of the BM at the superior disc margin and a gamma zone inferiorly. Due to the shifting of the BM opening in relationship to the peripapillary scleral flange opening, the ophthalmoscopically visible part of the optic disc decreases in size, so that “titled discs” appear to be small [63]. In eyes with a so called situs inversus papillae, in which the retinal vessel trunk abnormally exits into the nasal direction, BM is slightly overhanging at the temporal disc margin with a small gamma zone nasally. The histological equivalent of gamma zone is the absence of BM. Since the choriocapillaris forms with its basal membrane the outer layer of BM, the choriocapillaris and BM are firmly connected with each other. It implies that the gamma zone without BM neither has a choriocapillaris [65, 66]. In additional, Haller’s layer and Sattler’s layer of the choroid are usually missing in the gamma zone, which contains only some large vessels running from the short posterior ciliary arteries to the choroid. Some eyes show some choroidal tissue inside the gamma zone at the inner margin of the BM opening, perhaps since the shift of the BM opening was more marked than the accompanying shift of the choroidal opening.

Besides the gamma zone, the delta zone is present in the parapapillary region in highly myopic eyes (Figs. 1 and 2) [65, 66, 73]. It is characterized by an elongated and thinned peripapillary scleral flange. The latter is the continuation of the inner portion of the sclera, while the outer portion of the sclera continues into the optic nerve dura mater [69]. The peripapillary scleral flange is the anterior roof of the orbital CSF space and continues into the lamina cribrosa. At the merging zone of the peripapillary scleral flange with the lamina cribrosa, the collagen fibers of the peripapillary border tissue of the scleral flange as the continuation of the optic nerve pia mater run perpendicularly in a sagittal direction [48]. The scleral flange is the biomechanical anchor of the lamina cribrosa. The crisscrossing with the peripapillary border tissue may be of additional biomechanical importance since the peripapillary border tissue, coming from the pia mater and continuing through the peripapillary border tissue of the choroid to the end of BM may fixate the lamina cribrosa in a sagittal direction [48]. The elongation of the peripapillary scleral flange in highly myopic eyes may be due to the general, axial elongation-associated, elongation and thinning of the sclera. It may perhaps additionally be influenced by a potential backward pull of the optic nerve. Studies have suggested that in extremely high myopic eyes, the optic nerve may be too short to allow a full adduction so that a pull may be exerted mostly at the temporal and temporal inferior optic nerve border [74, 75]. The backward pull of the optic nerve in markedly axially elongated eyes may also be the reason for the development of suprapapillary choroidal cavitations that are usually located at the temporal to inferior disc border [76–79].

### Peripapillary arterial circle

The peripapillary arterial circle of Zinn-Haller is usually located at the merging line of the optic nerve dura mater with the posterior sclera i.e., the peripheral end of the peripapillary scleral flange [80, 81]. Since the scleral flange elongates in high myopia, the distance between the arterial circle and the lamina cribrosa nourished by the circle, enlarges [82, 83]. It may be an additional cause for an increased glaucoma susceptibility in highly myopic eyes [60, 61].

### Peripapillary border tissues

The optic disc is surrounded by the peripapillary ring [84]. It is a whitish band the width of which is not dependent on axial length. The peripapillary ring is the continuation of the optic nerve pia mater, which first continues into the peripapillary border tissue of the peripapillary flange (Elschnig), which continues into the peripapillary border tissue of choroid (Jacoby), and that finally connects to the end of BM [48]. Since the inner shells of the eye i.e., the uvea, BM, RPE and retina, are firmly connected with the sclera as the outer shell only at the scleral spur anteriorly and the peripapillary border of the choroid posteriorly, the latter has biomechanical importance. In axially elongated eyes, the choroidal border tissue increases in length parallel to a decrease in its thickness, so that its volume is independent of axial length [48]. The elongation of the peripapillary choroidal border tissue is the equivalent to the width of the parapapillary gamma zone and leads to an increase in the disc-fovea distance without affecting the length of BM in the macular region [43, 44, 47]. In some eyes, the adhesion of the choroidal border tissue on the end of the BM may rupture, so that the BM becomes loose and assumes a corrugated form, as can be seen upon histology and optical coherence tomography [85].

### Macula

Histological changes in the macular region of myopic eyes include a thinning of the choroid, most marked at the posterior pole, and less marked towards the fundus periphery [22, 86]. This choroidal thinning affects predominantly Haller’s and Sattler’s layer with the medium-sized and large choroidal vessels, while the choriocapillaris may remain mostly unchanged in its thickness and density with longer axial elongation [87]. The observation that the choriocapillaris density may not be strongly related to axial length may be explained by the firm attachment of the choriocapillaris to BM. It fits with the observations that the thickness and length of BM in the macular region as well as the density of the RPE cells and the retinal thickness in the macular region do not change with increasing axial length [11–13, 16, 17, 47]. In clinical category III of myopic maculopathy, patchy atrophies can be detected upon ophthalmoscopy in the extrafoveal region [88]. Histologically, and upon OCT-based histology, these patchy atrophies represent defects in BM, surrounded by a larger defect in the RPE cell layer [63, 89–93]. The reasons for the development of the macular BM defects has remained elusive so far. Longitudinal clinical and population-based studies have demonstrated that the BM defect enlarge in dependence of further axial elongation, female sex and higher degree of myopia [94–96]. Some of the BM defects may develop due to lacquer cracks, which histologically may represent linear defects in the RPE and BM. If the notion of an enlargement of BM in the fundus periphery associated with the axial elongation is valid, one may purport that the BM growth in the fundus periphery may not only lead to an increase in the globe size in the sagittal direction but also, however to a minor degree, to an eye enlargement in the horizontal and vertical directions [42]. This enlargement of the eye in the coronal directions may lead to an increased strain within BM, which first may lead to an enlargement of the BM opening of the optic nerve head [63]. If the BM opening enlargement is not sufficient to relax the BM strain, additional defects in BM may develop in the macular region. Interestingly, highly myopic eyes with a small BM opening as compared to highly myopic eyes with a large BM opening had a higher prevalence of macular BM defects, fitting with the notion that the enlargement of the BM opening of the optic nerve head protects against additional macular BM defects [63].

### Staphyloma

Another feature of highly myopic eyes are posterior staphylomas [97]. According to a recent histologic study, a staphylomatous region as compared to a corresponding region without sclera staphyloma was characterized by marked scleral thinning and spatially correlated BM defects, while the thickness and density of the choriocapillaris and RPE cell layer and the BM thickness did not differ significantly between the staphylomatous versus non-staphylomatous regions [98]. These findings supported the notion that a locally reduced scleral resistance against a backward pushing BM might have led to a local scleral outpouching. This scleral outpouching increased the scleral curvature length with a secondary stretching of BM with the sequel of a localized BM rupture and development of BM defects.

### Non-glaucomatous optic nerve damage

Besides an increased prevalence of a glaucomatous or glaucoma-like optic neuropathy, highly myopic eyes may also show an increased prevalence of non-glaucomatous optic nerve damage [99]. It may affect the retinal ganglion cell axons that are located in the papillo-macular region. It may be due to a parapapillary gamma zone-associated lengthening of the retinal nerve fibers, since gamma zone increases the disc-fovea distance [43, 45, 46]. Since the distance between the temporal superior arterial arcade and the temporal inferior arterial arcade is not affected by axial elongation in eyes without macular BM defects, the angle between the temporal arterial arcades decreases with longer axial length [100].

### Limitations

There are a couple of limitations to this review. First, it has to be emphasized that this work was focused on anatomical findings in highly myopic eyes, and that the discussion on the etiology of these changes was not the primary topic nor was it well balanced. It was mainly focused on the potential role of BM in the process of myopization and partially neglected other or complementing theories of the process of axial elongation. Neglecting in this review other hypotheses, such as those on the role of the choroid and sclera in myopization, does not indicate, that these hypotheses are invalid. To cite an example, it could also be the case that the axial elongation of the eye could be produced by other primary drivers other than BM, and that the globe elongation could induce stress on the RPE, which could then secondarily increase the production of BM in order to counteract the elongation. Such a process could perhaps also lead to a relative preservation of BM thickness in these highly elongated eyes. Second, it has remained unclear whether anatomical differences between normal eyes and myopic eyes were the cause or the effect of the process of axial elongation.

## Conclusions

High axial myopia is associated with a multitude of histological changes in the posterior hemisphere of the globe, most notably at the posterior pole and optic nerve head.

## Data Availability

Not applicable.

## Abbreviations

APON: Acquired pits of the optic nerve head
BM: Bruch’s membrane
CSF: Cerebrospinal fluid
OCT: Optical coherence tomography
RPE: Retinal pigment epithelium cells
